# Assessing the Impact of USMLE Step 1 Going Pass-Fail: A Brief Review of the Performance Data

**DOI:** 10.1055/s-0044-1800830

**Published:** 2024-12-24

**Authors:** Kevan English

**Affiliations:** 1 Department of Internal Medicine, University of Nebraska Medical Center College of Medicine, Omaha, Nebraska, United States

**Keywords:** USMLE, international medical graduates, medical students, pass-fail, ECFMG

## Abstract

On January 26, 2022, the United States Medical Licensing Examination (USMLE) Step 1 exam transitioned to a pass-fail grading system instead of the conventional three-digit score. This move was intended to decrease the emphasis on Step 1 scores and facilitate a more holistic approach by programs in the residency selection process. However, since the implementation of the new grading system, we have seen a lower passing percentage among all medical students, including U.S. MDs, DOs, and international medical graduates. In this article, we assess the USMLE Step 1 performance data since the change in scoring and some factors that may have contributed to the lower passing rates among all medical students.

## Introduction

In early 2022, the National Board of Medical Examiners (NBME) and the Federation of State Medical Boards made a monumental decision to shift the United States Medical Licensing Examination (USMLE) Step 1 grading to pass-fail from the traditional three-digit score. 1 This decision was additionally supported by the Education Commission for Foreign Medical Graduates (ECFMG) during the Invitational Conference on USMLE scoring. 1 2 It is important to note that less than 10% of representatives from the ECFMG attended this meeting. 3 This ruling, as expected, resulted in a stir among the medical community, with many questioning how it would affect medical students and the residency selection process. Two years later, we have data from the USMLE, which suggests lower passing rates among all medical students regardless of their classification as either U.S. or international medical graduates (IMGs). 4 5

**Fig. 1:**
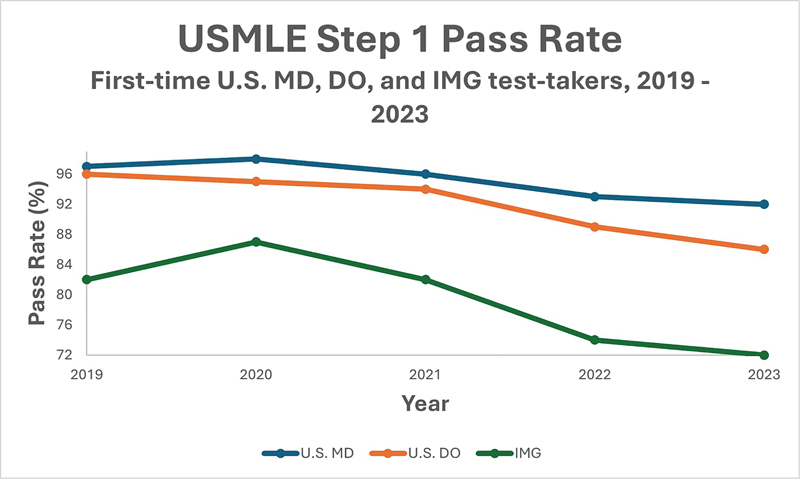
Line graph showing United States Medical Licensing Examination (USMLE) Step 1 pass rates among U.S. MD, U.S. DO, and international medical graduate (IMG) test-takers between 2019 and 2023.

Since the modification of the grading system to pass-fail in 2022, the Step 1 pass rate among U.S. MD students fell from 95% in 2021 to 91% in 2022. Similarly, the pass rate for U.S. DO seniors dropped to 89% in 2022 from 94% in 2021. The decrease in overall pass rate showed a similar trend among IMGs, with 82% of first-time test takers passing the test in 2021 down to 74% in 2022. The overall pass rate among all medical students decreased drastically from 88% in 2021 to 82% in 2022. 4 5 These numbers continue to decline among all first-time test takers, according to the recent data released by the USMLE for examinees tested in 2023 ( Fig. 1 ). 5 The passing rate saw a 1% drop to 90 in 2023, compared with 91% in 2022 among U.S. MDs. Similarly, U.S. DOs saw a 3% drop in first-time pass rate to 86% in 2023, compared with 89% the previous year. IMGs saw a 2% decline in pass rate down to 72% in 2023 compared with 74% in 2022. These numbers represent a significant increase in the number of medical students who have failed the exam since the grading system changed. While more analysis and research are needed to assess the reasons for the decline in the USMLE Step 1 first-time pass rates since the modification, we address some of the factors that may have contributed to this pattern below.

*A change in pass score* : Before 2022, the USMLE Step 1 exam was graded with a passing score of 194. 6 However, most students worked to earn a higher score to increase their chances of gaining their residency selections of choice. This changed in 2022, when the pass score shifted to 196. Although one would expect a drop in overall pass rates due to the increase in the score required to pass the exam, this alone would not explain the decline in overall pass rates from 88% in 2021 to 82% in 2022. In fact, the last time the passing standard was increased from 192 to 194 in 2018, the number of students who passed in the same year increased slightly compared with 2017. 5 6 7

*Coronavirus disease 2019 pandemic* : The events in 2020 brought significant disruption to the medical community, ultimately affecting medical student education and assessment. 8 9 This disruption continued into 2022 and most likely significantly impacted pass rates. 5 8 9 10 Since Step 1 is comprehensive, there may have been exam blocks that were adversely affected by the pandemic. 10

*Lack of effort in studying* : Naturally, due to the absence of the three-digit score and less pressure to perform well to increase the chance of gaining the residency position of choice, students allotted less effort in studying as this would not be reflected in a passing score report. 11 12 Medical students were also reported to have done fewer practice questions during exam preparation. 11

## Potential Solutions in Addressing Lower Pass Rates

Several potential solutions exist to improve USMLE Step 1 pass rates, especially among IMGs and minority students. The first and most crucial intervention starts with the medical committee and the curriculum at the respective medical schools. These curriculums should have a built-in number of USMLE Step 1 questions that students must complete with an appropriate target percentage correct as they navigate the first 2 years of medical school. This would allow students to gain the proper level of preparation regarding practice questions. Medical schools should also require students to take a certain number of USMLE-style practice tests, such as the NBME comprehensive basic science exams, with a target score before they can take the official test. 9 This would also enable students to build the stamina required to successfully pass the exam and address the underlying lack of study effort that came about after the change to pass-fail.

Another potential solution to addressing the lower pass rate is monitoring students closely to identify who might struggle. This is especially important at larger medical institutions. Medical schools should use examination scores, including their own and other preclinical exams, to identify students who may benefit from additional support. Schools can offer student support, including help from learning specialists, student affairs deans, wellness offices, and advisory deans, who can assist and provide a learning plan for them in preparation for the exam. 13

Lastly, while commonly overlooked, students with difficulties stemming from health or mental health stressors, psychosocial or family challenges, and social or situational stressors can encounter problems in Step 1 preparation. 14 Working with wellness offices and providing resources to support students with these challenges can foster successful performances on important exams such as the USMLE.

## Conclusion

In summary, the overall pass rates for USMLE Step 1 have declined since modifying the scoring system in 2022. While the increase in passing standards and the pandemic may have played a role in the decline of the passing rates, the decrease in the number of questions answered in preparation for the exam suggests that students may have become less focused on performing well on the exam although the overall drop in the pass rates in 2023 compared with 2022 were less compared with 2022 and 2021. Medical schools can implement measures to improve pass rates, such as more practice tests, more required USMLE questions, and mental health and social support.

## References

[1] Currie M Hammond C Martinez O P Lane-Cordova A Cook J The impact of United States Medical Licensing Examination Step 1 transitioning to pass/fail on medical student perception of research needed to match into one's preferred specialty Cureus 2024 16 04 e57395 38694632 10.7759/cureus.57395 PMC11061812

[2] Belovich A N Bahner I Bonaminio G USMLE Step-1 is going to pass/fail, now what do we do? Med Sci Educ 2021 31 04 1551 1556 34109056 10.1007/s40670-021-01337-4 PMC8177252

[3] Al-Akchar M Salih M Fanari Z USMLE step 1 pass/fail: the impact on international medical graduates Avicenna J Med 2021 11 01 40 41 33520788 10.4103/ajm.ajm_154_20 PMC7839260

[4] Yadav S Dekhne A Harikrishnan S The pass/fail effect: a longitudinal study of United States Medical Licensing Examination (USMLE) Step 1 performance over a decade Cureus 2023 15 07 e41702 37575720 10.7759/cureus.41702 PMC10415954

[5] USMLE. Performance data [Internet]. Accessed November 22, 2024 at:https://www.usmle.org/performance-data

[6] Keltner C Haedinger L Carney P A Bonura E M Preclinical assessment performance as a predictor of USMLE Step 1 scores or passing status Med Sci Educ 2021 31 04 1453 1462 34457984 10.1007/s40670-021-01334-7 PMC8368122

[7] Rayamajhi S Dhakal P Wang L Rai M P Shrotriya S Do USMLE steps, and ITE score predict the American Board of Internal Medicine Certifying Exam results? BMC Med Educ 2020 20 01 79 32183789 10.1186/s12909-020-1974-3 PMC7079442

[8] Cheloff A Z Bharadwa S A student perspective on taking the USMLE Step 1 during the COVID-19 pandemic Acad Med 2022 97 05 621 10.1097/ACM.0000000000004625 35476828

[9] Swan Sein A Daniel M Hauer K E Santen S A Educational and practical implications of Step 1 timing in the context of COVID-19 Med Sci Educ 2021 31 02 911 916 33777488 10.1007/s40670-021-01255-5 PMC7987737

[10] Southworth E Gleason S H COVID 19: a cause for pause in undergraduate medical education and catalyst for innovation HEC Forum 2021 33(1-2):125 142 33481144 10.1007/s10730-020-09433-5 PMC7821447

[11] Prasad S Perez C Carnevale K JF Medical students' perspective on the United States Medical Licensing Examination (USMLE) Step 1 transition to Pass/Fail MedEdPublish 2024 14 20 10.12688/mep.19975.3 PMC12322661 40765926

[12] Girard A O Qiu C Lake I V Chen J Lopez C D Yang R US medical student perspectives on the impact of a pass/fail USMLE Step 1 J Surg Educ 2022 79 02 397 408 34602379 10.1016/j.jsurg.2021.09.010

[13] Swan Sein A Dathatri S Bates T A Twelve tips on guiding preparation for both high-stakes exams and long-term learning Med Teach 2021 43 05 518 523 33032481 10.1080/0142159X.2020.1828570

[14] Rajkumar R P COVID-19 and mental health: a review of the existing literature Asian J Psychiatr 2020 52 102066 32302935 10.1016/j.ajp.2020.102066 PMC7151415

